# Simultaneous Quantitative Analysis of Polymorphic Impurities in Canagliflozin Tablets Utilizing Near-Infrared Spectroscopy and Partial Least Squares Regression

**DOI:** 10.3390/molecules31020230

**Published:** 2026-01-09

**Authors:** Mingdi Liu, Rui Fu, Guiyu Xu, Weibing Dong, Huizhi Qi, Peiran Dong, Ping Song

**Affiliations:** 1 College of Chemistry and Materials Science, Qinghai Minzu University, Xining 810007, China; 2 Key Laboratory of Resource Chemistry and Eco-Environmental Protection in Tibetan Plateau, State Ethnic Affairs Commission, Xining 810007, China; 3 Qinghai Provincial Key Laboratory of Nanomaterials and Technology, Qinghai Minzu University, Xining 810007, China

**Keywords:** canagliflozin, NIR, ACO, PLSR, quantitative mechanism

## Abstract

Canagliflozin (CFZ), a sodium–glucose cotransporter 2 (SGLT2) inhibitor, is extensively utilized in the management of type 2 diabetes. Among its various polymorphic forms, the hemi-hydrate (Hemi-CFZ) has been selected as the active pharmaceutical ingredient (API) for CFZ tablets due to its superior solubility. However, during the production, storage, and transportation of CFZ tablets, Hemi-CFZ can undergo transformations into anhydrous (An-CFZ) and monohydrate (Mono-CFZ) forms under the influence of environmental factors such as temperature, humidity, and pressure, which may adversely impact the bioavailability and clinical efficacy of CFZ tablets. Therefore, it is imperative to develop rapid, accurate, non-destructive, and non-contact methods for quantifying An-CFZ and Mono-CFZ content in CFZ tablets to control polymorphic impurity levels and ensure product quality. This research evaluated the feasibility and reliability of using near-infrared spectroscopy (NIR) combined with partial least squares regression (PLSR) for simultaneous quantitative analysis of An-CFZ and Mono-CFZ in CFZ tablets, elucidating the quantifying mechanisms of the quantitative analysis model. Orthogonal experiments were designed to investigate the effects of different pretreatment methods and ant colony optimization (ACO) algorithms on the performance of quantitative models. An optimal PLSR model for simultaneous quantification of An-CFZ and Mono-CFZ in CFZ tablets was established and validated over a concentration range of 0.0000 to 10.0000 w/w%. The resulting model, Y_An-CFZ/Mono-CFZ_ = 0.0207 + 0.9919 X, achieved an *R*^2^ value of 0.9919. By analyzing the relationship between the NIR spectral signals selected by the ACO algorithm and the molecular structure information of An-CFZ and Mono-CFZ, we demonstrated the feasibility and reliability of the NIR-PLSR approach for quantifying these polymorphic forms. Additionally, the mechanism of PLSR quantitative analysis was further explained through the variance contribution rates of latent variables (*LV*s), the correlations between *LV*s loadings and tablets composition, and the relationships between *LV* scores and An-CFZ/Mono-CFZ content. This study not only provides a robust method and theoretical foundation for monitoring An-CFZ and Mono-CFZ content in CFZ tablets throughout production, processing, storage, and transportation, but also offers a reliable methodological reference for the simultaneous quantitative analysis and quality control of multiple polymorphic impurities in other similar drugs.

## 1. Introduction

Polymorphism, which has attracted attention in the field of solid drug research and quality control, refers to the formation of different crystal structures of the same active pharmaceutical ingredient (API) under varying crystallization conditions [1,2,3]. These structural differences lead to significant variations in properties such as solubility (amorphous indomethacin shows higher solubility than its γ form), stability (ritonavir form II is more stable than form I), and bioavailability (the α form of azelnidipine exhibits better bioavailability compared to its β form), which significantly influence the efficacy and safety of drugs [1,2,3,4,5,6,7,8]. Therefore, accurate quantitative analysis of polymorphic impurities in drugs is essential for maintaining the quality and consistency of drug production.

Traditional analytical methods for quantifying polymorphic impurities in solid drugs, such as X-ray diffraction, thermal analysis, and vibrational spectroscopy, offer unique advantages but also have limitations like complex sample preparation, time-consuming processes, and high costs [9,10,11,12,13,14,15,16]. In recent years, near-infrared (NIR) spectroscopy has emerged as a promising technique due to its rapid, non-destructive nature, lack of reagent requirement, and capability for online monitoring [4,12,14,15,16]. NIR spectroscopy leverages the vibrational absorption characteristics of drug components in the near-infrared region to measure the absorption of near-infrared light by solid drug samples, thereby providing insights into hydrogen bonding and hydrogen-containing groups within the molecules [4,12,14,15,16,17]. However, NIR spectroscopy faces challenges such as severe spectral band overlap and weak signals, which hinder its application in accurately quantifying polymorphic impurities in solid drugs [4,12,14,15,16]. Thus, selecting an appropriate pretreatment method to preprocess the spectral data is crucial for achieving accurate quantitative analysis of polymorphic impurity content in solid drugs using NIR spectroscopy.

Effective pretreatment methods not only significantly improve the signal-to-noise ratio and analytical precision of spectral data but also provide a robust foundation for subsequent data analysis and modeling [4,5,11,12,15,16,18,19,20,21,22,23]. Extensive literatures have shown that techniques like Multivariate Scattering Correction (MSC), Standard Normal Variate transformation (SNV), Savitzky–Golay first derivative (SG1st), Savitzky–Golay second derivative (SG2nd), and Wavelet Transform (WT) effectively reduce interference factors such as instrument noise, baseline drift, and light scattering, thereby significantly enhancing the quality of spectral data [4,5,11,12,18,19,20,21,22,23]. Although spectral pretreatment methods significantly enhance the accuracy of quantitative analysis of chemical components in complex mixtures based on NIR spectra, a substantial amount of information in NIR spectra remains unrelated to the molecular structure of the sample components even after pretreatment [4,24]. Consequently, it is essential to employ an appropriate spectral data selection method to extract key information pertinent to the molecular structure of the sample components from the NIR spectral data.

In the 1990s, Marco Dorigo introduced the ant colony optimization (ACO) algorithm, which effectively addresses the optimal combination of NIR spectral bands by simulating the pheromone-based route-selection mechanism observed in ant foraging behavior [25,26,27,28,29]. This algorithm can be applied to model and predict the content of polymorphic impurities in solid drugs, offering advantages such as intelligent search, global optimization, and robust performance, which help reduce overlap and weak information in spectral data [25,26,27,28,29]. However, due to the complex composition of excipients in solid drugs, multicollinearity issues arise in the collected spectra, affecting the accurate quantification of polymorphic impurities.

To tackle this challenge, Partial Least Squares Regression (PLSR), which combines elements from Multiple Linear Regression (MLR), Principal Component Analysis (LVA), and Canonical Correlation Analysis (CCA), can mitigate multicollinearity by identifying latent structural relationships between independent and dependent variables [15,16,19,20,21,30,31]. By constructing latent variables as linear combinations of the original independent and dependent variables, PLSR enhances both the predictive and explanatory power of the model, thereby achieving rapid and precise prediction of impurity crystal content in drugs [15,16,19,20,21,30,31].

This study aims to optimize NIR spectral data using different pretreatment methods and the ACO algorithm to construct a PLSR model for rapid, non-destructive, and non-contact accurate quantitative analysis of the anhydrous (An-CFZ) and monohydrate (Mono-CFZ) contents in canagliflozin (CFZ) tablets. This approach not only enhances the efficiency of NIR analysis but also accurately determines the content of multiple polymorphic impurities in CFZ tablets simultaneously, offering an innovative solution for quality control of polymorphic drugs, thereby advancing the quality control practices in pharmaceutical research and production.

## 2. Materials and Methods

### 2.1. Materials and Samples Preparation

#### 2.1.1. Materials

Commercially available CFZ tablets were manufactured by blending Hemi-CFZ with excipients in a predetermined ratio and subsequently compressing them into tablets. During the tablet manufacturing process, Hemi-CFZ may undergo transformation into Mono-CFZ or An-CFZ due to environmental factors such as humidity, temperature, and pressure. These changes can potentially impact the safety and clinical efficacy of CFZ tablets. The Hemi-CFZ, An-CFZ, Mono-CFZ, and excipients utilized as standard substances in the experiment were all supplied by a partner, with a purity of ≥99.90%.

#### 2.1.2. Preparation of the Tablet Samples

**Figure 1:**
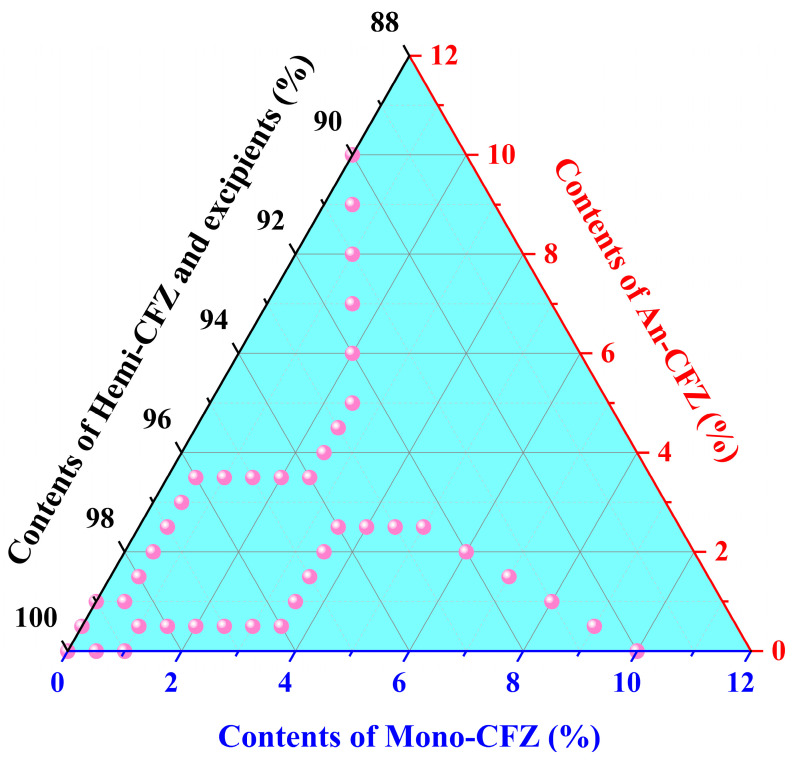
Samples used to establish and verify the quantitative models.

All standard substances were finely ground using an agate mortar and sieved through a 100-mesh screen. Based on the predetermined ratio of Hemi-CFZ to excipients found in commercially available CFZ tablets, Hemi-CFZ and excipients were thoroughly mixed. Subsequently, according to the ratios illustrated in Figure 1, corresponding weights of An-CFZ and Mono-CFZ were added, and a total of 2.0000 g of each CFZ tablet was prepared. All weighing procedures were conducted using a METTLER Toledo Electronic Balance (ME204T/02, Mettler Toledo Instrument (Shanghai) Co., Ltd., Shanghai, China).

### 2.2. Methods

#### 2.2.1. Powder X-Ray Diffraction

All PXRD patterns were obtained using the R-AXIS-RAPID powder diffractometer (Rigaku Co., Akishima, Japan) with Cu Kα radiation (λ = 1.5405 Å). The instrument was operated at 40 kV and 100 mA. All tests were performed under ambient conditions, with a step size of 0.02° and a scanning rate of 8°/min over the 2*θ* range of 2 to 40°. Each sample underwent three PXRD analyses. To maintain consistency and accuracy, all tests were conducted under standardized settings, ensuring reproducibility of the results. The specified operating parameters and environmental conditions were strictly adhered to throughout the analysis process.

#### 2.2.2. Near Infrared Spectroscopy

All NIR spectra were recorded using an FT-NIR spectrometer (Thermo Fisher Scientific Antaris II, Waltham, MA, USA). Each spectrum was the average of 16 scans covering the spectral range of 10,000–4000 cm^−1^ (wavenumber interval was 4 cm^−1^) at a resolution of 8 cm^−1^. All standard substances were scanned three times, and the average spectrum was calculated and used as their spectra. For each calibration sample, three consecutive scans were performed, and the average spectrum was calculated. Subsequently, this sample was removed and replaced, and the above process was repeated twice, yielding three sets of parallel spectral data. To ensure data reliability and accuracy, all measurements were conducted under standardized conditions. The repeated scans provided robust datasets, enhancing the precision of the spectral information obtained. This approach facilitated reliable and reproducible results for further analysis.

### 2.3. Pretreatment of Spectra Data

In accordance with the division principles for the calibration and validation sets of small-scale datasets and the actual needs of the partners, the NIR spectra, represented by a 129 × 1550 spectral data matrix, were specifically partitioned into a calibration set (a 41 × 3 × 1550 spectral matrix) and a verification set (a 6 × 1550 spectral matrix). The calibration set consisted of 41 samples, each of which was taken three times, yielding an average spectrum as described in Section 2.2.2. Whereas the verification set included 6 samples taken from the same concentration level with the calibration set and re-prepared, each was scanned three times consecutively, and the average spectrum was taken. These datasets were used to develop and validate quantitative models aimed at determining the concentrations of An-CFZ and Mono-CFZ in CFZ tablets.

To optimize spectral bands selection and reduce extraneous information caused by scattering effects, variations in light path due to non-uniform particle distribution, and differences in particle sizes, the ACO algorithm was employed alongside various preprocessing techniques, including MSC, SNV, SG1st, SG2nd, and WT. Orthogonal experiments were designed to investigate the influence of the impact of parameters like ant numbers, the number of selected spectral bands, pheromone evaporation rates, and pretreatment methods on the performance of the PLSR model. These techniques ensured that the processed spectral data accurately reflected their intrinsic characteristics.

Subsequently, PLSR analysis was conducted using MATLAB 2024a to establish robust PLSR models for NIR analysis. Linear regression data processing was performed using Origin 9.1 software. Model performance was evaluated based on the Correlation Coefficient (*R*^2^), Root Mean Square Error of Calibration (*RMSEC*), Cross-Validation (*RMSECV*), and Prediction Error (RMSEP). These metrics were calculated based on Equations (1) and (2).

(1) $$R^{2}=1-\left(\sum_{i=1}^{n}{\left(y_{i}-{\hat{y}}_{i}\right)}^{2}/\sum_{i=1}^{n}{\left(y_{i}-\bar{y}\right)}^{2}\right)$$

(2) $$RSME=\sqrt{\sum_{i=1}^{n}{\left(y_{i}-{\hat{y}}_{i}\right)}^{2}/n}$$

where $y_{i}$, ${\hat{y}}_{i}$, $\bar{y}$, and n in Equations (1) and (2) represent the theoretical value, calculated value, averaged value, and the number of samples, respectively.

### 2.4. Validation of Developed Models

To validate the accuracy of the correction models, NIR was employed to analyze samples with known An-CFZ (0.5000%, 1.5000%, 2.5000%, 3.5000%, 4.5000%, and 5.0000% *w*/*w*) and Mono-CFZ content (0.5000%, 1.5000%, 2.5000%, 3.5000%, 4.5000%, and 5.0000% *w*/*w*), and the established PLSR model was used for prediction. Furthermore, the precision, repeatability, and stability of NIR were assessed using Mono-CFZ and An-CFZ content at a concentration level of 4.5000% *w*/*w*. The Relative Standard Deviation (*RSD*%) was calculated for the PLSR model according to Equation (3), whereas the Limit of Detection (*LOD*) and Limit of Quantification (*LOQ*) were determined for the PLSR model using Equations (4) and (5), respectively.

(3) $$RSD=\left(\sqrt{\sum_{i=1}^{n}{\left({\hat{y}}_{i}-\bar{y}\right)}^{2}/n-1}/\bar{y}\right)\times 100\%$$

(4) LOD=3.3σ/s

(5) LOQ=10σ/s

where ${\hat{y}}_{i}$, $\bar{y}$, n, σ, and s in Equations (3)–(5) represent the predicted content values, the average of the predicted content values of the samples, the number of the samples, the standard deviation of predicted content values, and the slope of the correlation curve, respectively.

## 3. Results and Discussion

### 3.1. Characterization of Different Forms of CFZ

**Figure 2:**
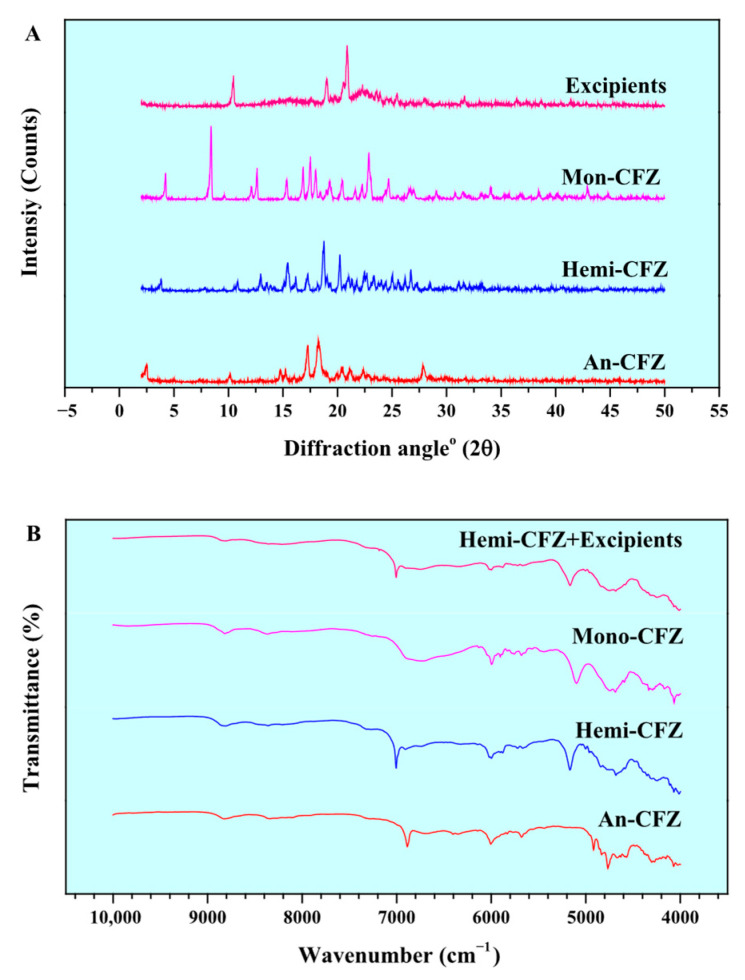
PXRD patterns and NIR spectra of standard substances. (**A**) PXRD patterns of An-CFZ, Hemi-CFZ, Mono-CFZ, and excipients; (**B**) NIR spectra of An-CFZ, Hemi-CFZ, Mono-CFZ, and Hemi-CFZ + excipients.

Figure 2 presents the PXRD patterns and NIR spectra of An-CFZ, Hemi-CFZ, Mono-CFZ, and excipients. In Figure 2A, 2.4°, 10.1°, 17.2°, 18.1°, 27.8° 2*θ* were considered as the characteristic peaks of An-CFZ, and 3.8°, 10.9°, 13.0°, 15.5°, 16.2°, 17.3°, 18.8°, 20.3° 2*θ* and 4.1°, 8.3°, 12.0°, 12.5°, 15.3°, 16.8°, 17.4°, 17.9°, 20.4°, 22.8° 2*θ* were that of Hemi-CFZ and Mono-CFZ, respectively. The PXRD characteristic peaks of the three CFZ crystal forms were in complete agreement with previously reported data, confirming that the An-CFZ, Hemi-CFZ, and Mono-CFZ samples provided by the partner meet the required standards for crystal structure and purity [14,24,32].

In Figure 2B, the NIR characteristic peaks of the three CFZ crystal forms align with the findings of Liu et al., demonstrating significant differences among the crystal forms [14,24]. This suggested the potential of using NIR spectroscopy to quantitatively analyze polymorphic impurity content in CFZ tablets. However, Figure 2B also revealed that the NIR spectra of Hemi-CFZ and excipients exhibit characteristics highly similar to those of An-CFZ and Mono-CFZ, which may complicate the quantitative analysis of polymorphic impurity content in CFZ tablets using NIR spectra.

### 3.2. Construction of NIR Spectral Database

**Figure 3:**
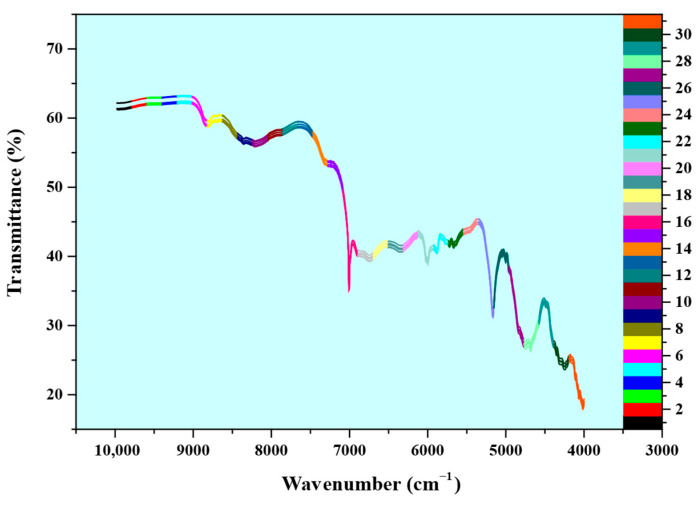
ATR-FTIR spectra of 31 submatrices. Different colors on the curves represent different spectral band numbers.

The NIR spectra of 41 different CFZ tablet samples, each containing varying amounts of An-CFZ and Mono-CFZ (with each sample measured in triplicate and the average value used as the primary spectral data), were utilized for the quantitative analysis of An-CFZ, Mono-CFZ, and total polymorphic impurity (Mono-CFZ + An-CFZ) contents in CFZ tablets, as illustrated in Figure S1. The calibration and validation sets were divided into 31 submatrices to construct a new NIR spectral database. This database was employed to select the optimal spectral bands for the PLSR model using the ACO algorithm, as shown in Figure 3.

Figure 3 presents the NIR spectra of CFZ tablet samples with five different concentrations of An-CFZ (10.0000%, 5.0000%, 1.5000%, 2.5000%, and 0.0000%) and Mono-CFZ (0.0000%, 2.5000%, 3.5000%, 5.0000%, and 10.0000%). Each spectrum represents the average of nine parallel measurements.

### 3.3. Selection and Pretreatment of NIR Spectra

**Figure 4:**
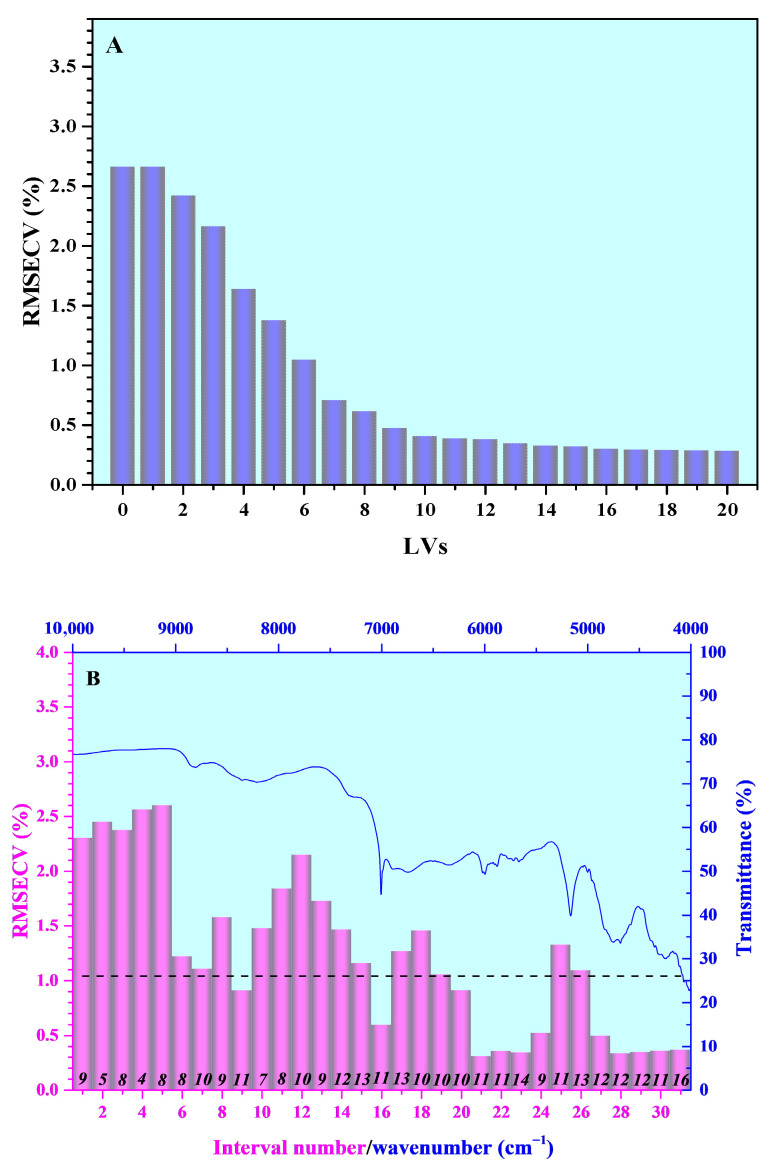
The *RMSECV* of PLSR models. (**A**) *RMSECV* vs. *LV*s of global PLSR model and (**B**) *RMSECV* of IPLS interval models. The dotted line represents the *RMSECV* for the global PLSR model, and the italic numbers indicate optimal *LV*s of interval models. The blue line represents the NIR spectra of Hemi-CFZ.

Raw NIR spectra of CFZ tablets frequently contain significant noise and redundant information, which could compromise the accuracy and efficiency of quantitative analysis for An-CFZ and Mono-CFZ. To mitigate this issue, we developed PLSR models using both the global NIR spectrum and spectra divided into 31 sub-intervals (taking 50 wavenumber sampling points as a sub-interval, the width of each sub-interval was approximately 200 cm^−1^). The results are presented in Figure 4.

As shown in Figure 4A, when the number of latent variables (*LV*s) was set to 5, the average *RMSECV* value of the global spectral PLSR model reached 1.0432%. Although further increasing the number of *LV*s (*N*) slightly decreases the average *RMSECV* value, the improvement is marginal and may increase the risk of overfitting. From Figure 4B, it is evident that the average *RMSECV* values of PLSR models based on sub-intervals 9, 16, 20, 21, 22, 23, 24, 28, 29, 30, and 31 are lower than those of the global spectrum PLSR model, indicating that the information within these sub-intervals is critical for the quantitative analysis of polymorphic impurities in CFZ tablets.

**Figure 5:**
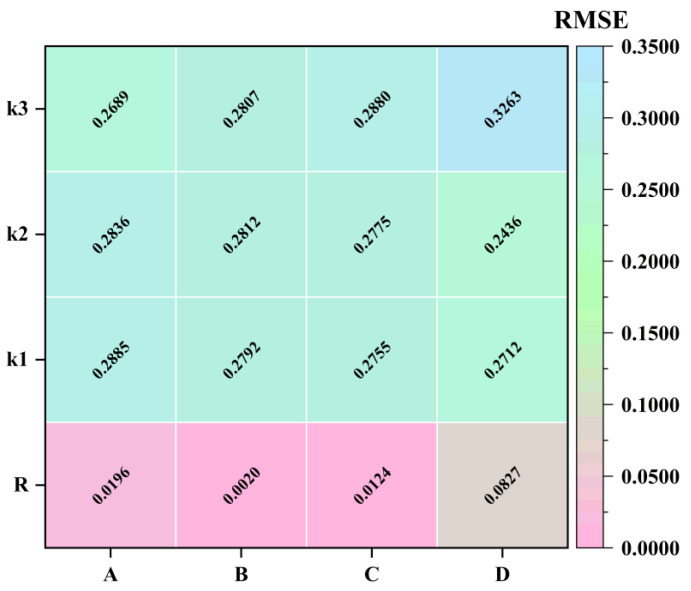
Results of orthogonal experiment. k1, k2, and k3 represent the average *RMSEP* values of experiments for each factor at each level, and R represents the range values of k1, k2, and k3 for each factor. *A*, *B*, *C*, and *D* represent the number of ants, the pheromone evaporation rates, the number of selected spectral bands, and pretreatment methods, respectively.

Therefore, prior to establishing the optimization PLSR quantitative analysis model, we employed the ACO algorithm along with various pretreatment methods to select and pretreat the raw NIR spectra. Key parameters of the ACO algorithm include the number of ants (*A*), the pheromone evaporation rate (*B*), and the number of selected spectral bands (*C*). Among them, *A* is directly associated with the exploration ability and computational efficiency, *B* functions as the lever for balancing exploration and exploitation, and *C* determines the complexity of the space and the difficulty of the search. Since the number of iterations of the ACO algorithm can theoretically be infinite, it has minimal impact on spectral band selection. Various pretreatment methods (*D*) effectively reduce noise in the raw NIR spectra, thereby enhancing the performance of the PLSR quantitative model. To optimize the selection and pretreat of the raw NIR spectra, a four-factor, three-level orthogonal experiment (as shown in Table 1) was designed. Through single-factor experiments, the optimal levels for each factor were chosen, and the average *RMSEP* of the PLSR model served as the evaluation criterion in all experiments. The orthogonal experimental results are illustrated in Figure 5.

As indicated by the extreme value (*R*) in Figure 5, the influence of the pretreatment method (*D*), number of ants (*A*), number of selected bands (*C*), and pheromone evaporation rate (*B*) on the performance of the PLSR model decreases in that order. Specifically, the pretreatment method has the most significant impact on PLSR model performance, followed by the number of ants and the number of selected bands, with the pheromone evaporation rate having the least effect. Therefore, the ranking of each factor’s influence on PLSR model performance is *D* > *A* > *C* > *B*.

Moreover, Figure 5 illustrates the changes in *RMSEP* values under different conditions: the *RMSEP* value was minimized when the preprocessing method was SG2nd, the number of ants was 300, the number of selected spectral bands was 5, and the pheromone evaporation rate was 0.3. This indicates that the combination *D*_2_*A*_3_*C*_1_*B*_1_ provides the best experimental conditions for optimal PLSR model performance.

Additionally, considering that the analysis efficiency of each experiment decreases with the number of ACO algorithm iterations, it was found that the highest quantitative analysis efficiency occurs under the following conditions: SG2nd pretreatment of the raw NIR spectrum, with 300 ants, 10 selected spectral bands, and a pheromone evaporation rate of 0.3. This may be attributed to the increased number of spectral bands aiding in more effectively distinguishing An-CFZ and Mono-CFZ components in CFZ tablets. Consequently, *D*_2_*A*_3_*C*_2_*B*_1_ represents the optimal combination of experimental conditions.

### 3.4. Establishment of PLSR Model

**Figure 6:**
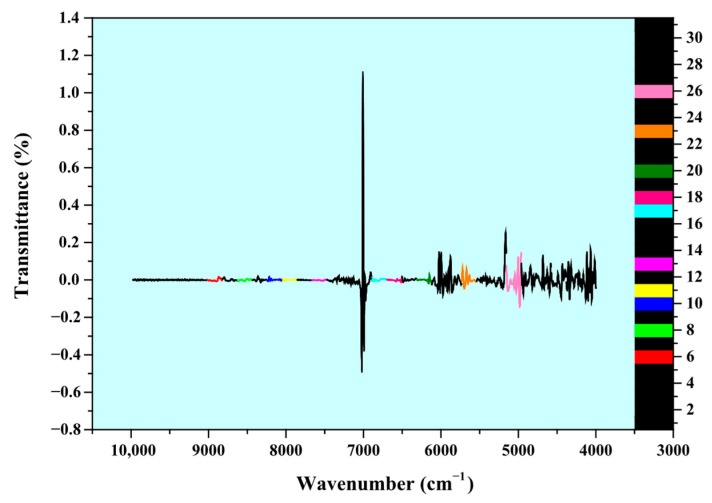
NIR spectra of 31 submatrices. The black portions on the curves represent spectral bands not selected by the ACO algorithm.

Based on the optimal conditions determined above, the ACO algorithm selected the following spectral bands to establish the PLSR model: 20, 17, 26, 11, 18, 10, 8, 13, 6, and 23. The results after SG2nd preprocessing are shown in Figure 6.

**Figure 7:**
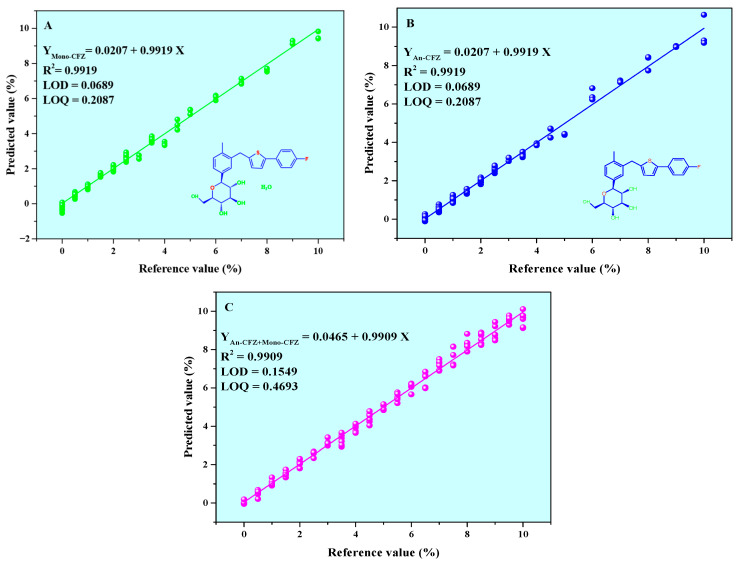
Calibration curve of Mono-CFZ, An-CFZ, and total polymorphic impurities. (**A**) Calibration curve of Mono-CFZ; (**B**) Calibration curve of An-CFZ; (**C**) Calibration curve of total polymorphic impurities.

The *N* value in the PLSR model is a critical parameter influencing model performance. Therefore, the *RMSEC* of the PLSR model’s predicted values was used as an evaluation metric to analyze the impact of different *N* values on model performance, with specific results shown in Table 2. As indicated in Table 2, when *N* ≤ 4, *RMSEC* (An-CFZ, Mono-CFZ, total polymorphic impurities, average) was ≥0.3000%, despite the high prediction efficiency of the model. Conversely, when *N* > 5, *RMSEC* (An-CFZ, Mono-CFZ, total crystalline impurity, average) was less than 0.3000%, leading to significantly improved prediction accuracy but reduced prediction efficiency. After a comprehensive evaluation of prediction accuracy, efficiency, and stakeholder requirements, *N* = 5 was chosen as the optimal solution. The final Calibration curve between the reference values and predicted values was Y_An-CFZ/Mono-CFZ_ = 0.0207 + 0.9919 X, *R*^2^ = 0.9919, and the Calibration curve of An-CFZ, Mono-CFZ, and total polymorphic impurities is illustrated in Figure 7.

### 3.5. Verification and Evaluation of the PLSR Model

The optimal PLSR model established using the ACO algorithm selected spectral bands was validated using a series of CFZ tablet samples with known Mono-CFZ, An-CFZ, and total polymorphic impurity contents (as shown in Table 3). NIR spectra of these samples were collected and pretreated using the SG2nd method. The spectra were reconstructed based on specific spectral bands selected by the ACO algorithm (20, 17, 26, 11, 18, 10, 8, 13, 6, and 23). The optimal PLSR model was then used to predict the contents of Mono-CFZ, An-CFZ, and total polymorphic impurities in the known tablet samples to evaluate the model’s prediction accuracy. The results are summarized in Table 3. To further validate the method’s effectiveness, particular attention was given to the data collection for samples with Mono-CFZ, An-CFZ, and total polymorphic impurity contents of 4.5000%, 4.5000%, and 9.0000% *w*/*w*, respectively. Additionally, the precision, repeatability, and stability results are also presented in Table 3.

As illustrated in Table 3, the optimal PLSR model accurately predicted the content of An-CFZ, Mono-CFZ, and total polymorphic impurities in six known CFZ tablet samples. Specifically, the mean prediction errors were 0.0141%, 0.0099%, and 0.0550% for An-CFZ, Mono-CFZ, and total polymorphic impurities, respectively. These low error rates indicate a high degree of consistency between the predicted and reference values, thereby confirming the robust predictive performance of the PLSR model.

### 3.6. Discussion on Quantitative Mechanism of PLSR Model

As illustrated in Figure 4B, the sub-interval PLSR model that divides the original NIR spectrum into 31 sub-intervals (taking 50 wavenumber sampling points as a sub-interval, the width of each sub-interval was approximately 200 cm^−1^) and establishes them separately shows lower *RMSE* values for several sub-intervals compared to the global-spectrum PLSR model. Specifically, the sub-intervals with lower *RMSE* values are numbered 21, 28, 23, 29, 22, 30, 31, 27, 24, 16, and 9, with sub-interval 21 achieving the lowest *RMSECV* of 0.3031%. In contrast, the optimal PLSR model established by selecting spectral bands using the ACO algorithm has modeling bands at positions 20, 17, 26, 11, 18, 10, 8, 13, 6, and 23 (as shown in Table 4), resulting in an *RMSE* of 0.2411%. This indicates superior performance over both the global-spectrum PLSR model and the individual sub-interval PLSR models. Notably, there is a discrepancy between the sub-intervals selected by the ACO algorithm and those with lower *RMSE* values compared to the global-spectrum model. Therefore, it is essential to further investigate the relationship between the spectral information selected by the ACO algorithm and the structural characteristics of Mono-CFZ and An-CFZ to better understand the quantitative analysis mechanism of the optimal PLSR model.

As illustrated in Table 4, the NIR spectral subintervals selected by the ACO algorithm are predominantly located within the 9010–7471 cm^−1^ and 6888–4964 cm^−1^ regions. Specifically, the 9010–7471 cm^−1^ region primarily captures the fundamental vibrational information of various chemical bonds in the molecule, aiding in distinguishing excipients from active ingredients (An-CFZ, Hemi-CFZ, and Mono-CFZ) in CFZ tablets, such as the overtone information of C=O in the aliphatic hydrocarbons at 8621 cm^−1^ [17]. Meanwhile, the 6888–4964 cm^−1^ region provides information on the fundamental frequency vibration’s overtones and combination information of O-H in crystalline water, hydroxyl and hydrogen bonds, as well as the combination frequencies of O-H stretching vibration and H-O-H bending vibration of water molecules, effectively highlighting subtle differences among An-CFZ, Hemi-CFZ, and Mono-CFZ. For instance, the peaks at 6913 cm^−1^ and 6811 cm^−1^ are likely attributed to the fundamental frequency vibration’s overtones and combinations information of hydroxyl groups and hydrogen bonds in Hemi-CFZ within CFZ tablets [17,33,34,35]. Furthermore, the peak at 5099 cm^−1^ may indicate the combination frequencies of O-H stretching and H-O-H bending vibrations of water molecules in Hemi-CFZ/Mono-CFZ of CFZ tablets [35].

In conclusion, the fundamental frequency information of various chemical bonds, along with the vibrational overtones and combination information of hydrogen bonds and hydrogen-containing groups observed through NIR spectra, provides a valuable opportunity and theoretical foundation for using the ACO algorithm to select the NIR global-spectrum for the quantitative analysis of An-CFZ, Mono-CFZ, and total polymorphic impurities in CFZ tablets.

**Figure 8:**
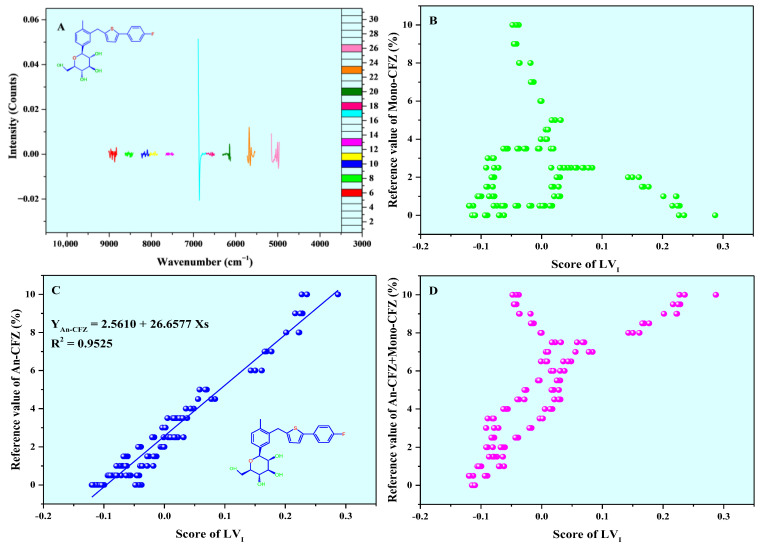
Loadings (**A**) and scores of *LV*_I_ (**B**–**D**). The color curves in A represent the ACO algorithm-selected spectral bands, and the white represents the ACO algorithm-not-selected spectral bands. (**B**–**D**) represent the relationship between the scores of *LV*_I_ and the reference values of Mono-CFZ, An-CFZ, and total polymorphic impurity (Mono-CFZ + An-CFZ) content, respectively.

The PLSR model constructed using NIR spectra, which was pretreated with SG2nd and selected by the ACO algorithm, is most suitable for the quantitative analysis of An-CFZ and Mono-CFZ content in CFZ tablets. The study further explores the mechanism of this model in quantifying these two components within CFZ tablets. Figure 8 illustrates the loadings and scores of *LV*_I_, where *LV*_I_ accounts for 20.9512% of the molecular structure information and 50.0589% of the polymorphic impurities content information of CFZ tablets (Figure 8A). A robust quantitative relationship was established between scores of *LV*_I_ and the An-CFZ content in CFZ tablets, with the correction curve expressed as Y_An-CFZ_ = 2.5610 + 26.6577 Xs and *R*^2^ = 0.9525 (Figure 8C), indicating that *LV*_I_ primarily captures An-CFZ-related information in CFZ tablets.

**Figure 9:**
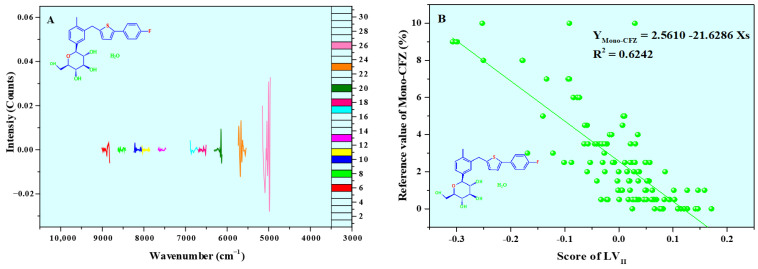

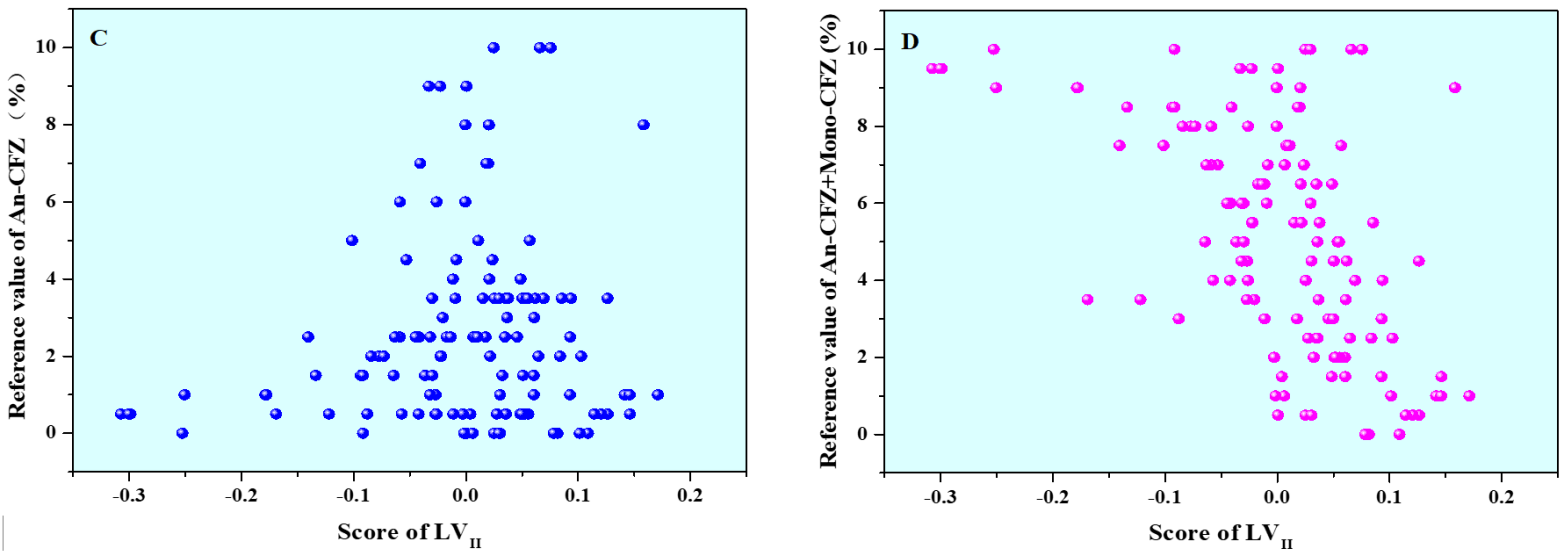
Loadings (**A**) and scores of *LV*_II_ (**B**–**D**). The color curves in A represent the ACO algorithm-selected spectral bands, and the white represents the ACO algorithm-not-selected spectral bands. (**B**–**D**) represented the relationship between the scores of *LV*_II_ and the reference values of Mono-CFZ, An-CFZ, and total polymorphic impurity (Mono-CFZ + An-CFZ) content, respectively.

*LV*_II_ explains 36.0995% of the molecular structure information and 31.5720% of the polymorphic impurity content information of the CFZ tablet (Figure 9A). A strong quantitative relationship was established between its scores and the Mono-CFZ content in CFZ tablet samples, with the correction curve given by Y_Mono-CFZ_ = 2.5610 − 21.6286Xs and *R*^2^ = 0.6242 (Figure 9B), indicating that *LV*_II_ primarily captures Mono-CFZ-related information in the CFZ tablets.

*LV*_III_, *LV*_IV_, and *LV*_V_ account for 13.3664%, 12.7601%, and 0.9839% of the molecular structure information, and 16.5380%, 0.2287%, and 0.7531% of the polymorphic impurity content information of the CFZ tablet, respectively (Figures S2–S4). Although these *LV*s explain a smaller proportion of the information, they still contribute significantly to enhancing model performance.

These five *LV*s explain 84.1611% of the molecular structure information and 99.0517% of the polymorphic impurity content information in CFZ tablets, demonstrating that the model effectively utilizes nearly all relevant information from the reconstructed NIR spectra. This model is reliable for the quantitative analysis of An-CFZ and Mono-CFZ in CFZ tablets. Specifically, *LV*_I_ is primarily used for the quantitative analysis of An-CFZ, while *LV*_II_ is mainly used for the quantitative analysis of Mono-CFZ. The collaborative effort of these five *LV*s enhances the accuracy and precision of the quantitative analysis of An-CFZ and Mono-CFZ.

Compared with the study published by Liu et al. in 2024 [24], this study significantly improved model performance and further clarified the feasibility, reliability, and mechanism of using NIR spectroscopy for the simultaneous quantitative analysis of An-CFZ and Mono-CFZ contents in CFZ tablets.

## 4. Conclusions

The aim of this study was to develop a rapid, accurate, non-destructive, and non-contact method for the simultaneous quantitative analysis of multiple polycrystalline impurities in drugs based on NIR spectroscopy, and to investigate its feasibility, reliability, and mechanism, thereby effectively controlling the content of polymorphic impurities and ensuring drug quality during production.

Early studies have shown that PLSR models based on the original global-spectrum exhibit and 31 sub-intervals have poor performance. Therefore, we conducted various pretreatments of NIR spectra within 31 sub-intervals (the range of 10,000 to 4000 cm^−1^) and employed the ACO algorithm to select the most suitable spectral bands, and established the optimal PLSR model for simultaneous quantitative analysis of An-CFZ and Mono-CFZ in CFZ tablets.

The reliability of the model in research was evaluated using N, *LV* variance contribution rate, cumulative variance contribution rate, and the relationship between *LV* scores and the content of An-CFZ and Mono-CFZ. Additionally, the relationship between the spectral information of the optimal modeling band selected by the ACO algorithm and the characteristic chemical bonds, hydrogen bonds, and hydrogen-containing groups in An-CFZ and Mono-CFZ molecules revealed the feasibility and mechanism of simultaneous quantitative analysis of these components in CFZ tablets using NIR spectroscopy.

This study provides theoretical support for the simultaneous quantitative analysis of multiple polymorphic impurities during the production, storage, and transportation of CFZ tablets, providing a reference method for quality control of CFZ tablets and a reliable basis for the quantitative analysis and quality control of other similar drugs. However, it cannot be denied that further exploration and discussion are still needed in the analysis of intact commercial tablets and the transferability of the method.

## Figures and Tables

**Table 1:** Factors and levels of orthogonal experiment.

Experiment Number	*A* ^a^	*B* ^a^	*C* ^a^	*D* ^a^
1	100	0.3	5	SG1st
2	100	0.5	10	SG2nd
3	100	0.7	15	SG2nd + WT
4	200	0.3	10	SG2nd + WT
5	200	0.5	15	SG1st
6	200	0.7	5	SG2nd
7	300	0.3	15	SG2nd
8	300	0.5	5	SG2nd + WT
9	300	0.7	10	SG1st

^a^ *A* represents the number of ants, *B* represents the pheromone evaporation rate, *C* represents the number of spectral bands, and *D* represents the pretreatment method.

**Table 2:** Effects of different *LV* numbers on PLSR model performance.

N	RMSEC			
	An-CFZ	Mono-CFZ	An-CFZ + Mono-CFZ	Average
1	2.4550	0.7807	2.0416	1.7591
2	0.2573	0.4495	0.5637	0.4235
3	0.2348	0.4166	0.4922	0.3812
4	0.2313	0.2427	0.3225	0.2655
5	0.2215	0.2214	0.2803	0.2411
6	0.2209	0.1886	0.2594	0.2230
7	0.1780	0.1636	0.2242	0.1886
8	0.1652	0.1424	0.1813	0.1630
9	0.1377	0.1458	0.1655	0.1497
10	0.1313	0.1268	0.1502	0.1361
15	0.0777	0.0758	0.0806	0.0780
20	0.0410	0.0567	0.0519	0.0499
25	0.0273	0.0269	0.0240	0.0260

**Table 3:** Validation results of the PLSR model.

Samples	Mono-CFZ (%)		An-CFZ (%)		Total Polymorphic Impurity (%)	
	Reference	Predicted	Reference	Predicted	Reference	Predicted
V1 ^a^	0.5000	0.4923	0.5000	0.4927	1.0000	0.9783
V2 ^a^	1.5000	1.4788	1.5000	1.4801	3.0000	2.9789
V3 ^a^	2.5000	2.4971	2.5000	2.4984	5.0000	5.0331
V4 ^a^	3.5000	3.5052	3.5000	3.5047	7.0000	6.8749
V5 ^a^	4.5000	4.4866	4.5000	4.5025	9.0000	9.1205
V6 ^a^	5.0000	4.9657	5.0000	4.9769	10.0000	9.9915
Precision (*RSD*%)	4.5000	1.1047	4.5000	0.9978	9.0000	1.2369
Repeatability (*RSD*%)	4.5000	1.1123	4.5000	1.0114	9.0000	1.2517
Stability (*RSD*%)	4.5000	1.2131	4.5000	1.1327	9.0000	1.2434

^a^ V1, V2, V3, V4, V5 and V6 are the samples used to validate the PLSR model.

**Table 4:** Wavenumber region corresponding to the spectral band number selected by the ACO algorithm to establish the optimal PLSR model.

Spectral Band Number	Wavenumber	Spectral Band Number	Wavenumber
20	6310–6121 cm^−1^	10	8238–8049 cm^−1^
17	6888–6699 cm^−1^	8	8624–8435 cm^−1^
26	5153–4964 cm^−1^	13	7660–7471 cm^−1^
11	8046–7857 cm^−1^	6	9010–8821 cm^−1^
18	6696–6507 cm^−1^	23	5731–5542 cm^−1^

## Data Availability

The original contributions presented in this study are included in the article and Supplementary Materials. Further inquiries can be directed to the corresponding author.

## Supplementary Materials

The following supporting information can be downloaded at https://www.mdpi.com/article/10.3390/molecules31020230/s1. Table S1: Samples used to establish the An-CFZ/Mono-CFZ content quantitative models; Figure S1: NIR spectral of the CFZ tablet samples; Figure S2: Loadings (A) and scores of *LV*_III_ (B–D); Figure S3: Loadings (A) and scores of *LV*_IV_ (B–D); Figure S4: Loadings (A) and scores of *LV*_V_ (B–D).
