# Supplementary material for: Simultaneous Quantitative Analysis of Polymorphic Impurities in Canagliflozin Tablets Utilizing Near-Infrared Spectroscopy and Partial Least Squares Regression
Source: Molecules. 2026 Jan 9;31(2):230. doi: 10.3390/molecules31020230 (PMC12843906; doi:10.3390/molecules31020230)
Supplement: Supplementary file 1 [file molecules-31-00230-s001.zip › molecules-3997673-supplementary.pdf]

Simultaneous Quantitative Analysis of Polymorphic Impurities in Canagliflozin Tablets Utilizing Near-Infrared Spectroscopy and Partial Least Squares Regression

Mingdi Liu[1, 2, 3, *], Rui Fu[1, 2], Guiyu Xu[1, 2], Weibing Dong[1, 2], Huizhi Qi[1, 2], Peiran Dong [1, 2], Ping Song[1, 2, 3]

[1] College of Chemistry and Materials Science, Qinghai Minzu University, Xining 810007, China

[2] Key Laboratory of Resource Chemistry and Eco-environmental Protection in Tibetan Plateau, State Ethnic Affairs Commission, Xining 810007, China

[3] Qinghai Provincial Key Laboratory of Nanomaterials and Technology, Qinghai Minzu University, Xining 810007, China

***Corresponding author:**

Mingdi Liu, Ph.D.

Associate Professor

College of Chemistry and Materials Science, Qinghai Minzu University, 3 Bayi Rd., Chengdong District, Xining, Qinghai 810007, China

Tel: +86-971-8173864

E-mail: liumingdi@tju.edu.cn (Mingdi Liu)

SUPPLEMENTARY INFORMATION

Samples used to establish the An-CFZ/Mono-CFZ content quantitative models were shown in Table S1.

NIR spectral of the CFZ tablet samples were shown in Figure S1.

Loadings (A) and scores of LV_{III} (B, C, D) was shown in Figure S2.

Loadings (A) and scores of LV_{IV} (B, C, D) was given in Figure S3.

Loadings (A) and scores of LV_{V} (B, C, D) was shown in Figure S4.

Table S1. Samples used to establish the An-CFZ/Mono-CFZ content quantitative models.

Number of samples	Content of An-CFZ （%）	Content of Mono-CFZ (%)	Content of Hemi-CFZ + excipients (%)	Content of total impurities (%)
1	10.0	0.0	90.0	10.0
2	1.0	0.0	99.0	1.0
3	0.5	0.0	99.5	0.5
4	0.0	0.0	100.0	0.0
5	9.0	0.5	90.5	9.5
6	3.5	0.5	96.0	4.0
7	3.0	0.5	96.5	3.5
8	2.5	0.5	97.0	3.0
9	2.0	0.5	97.5	2.5
10	1.5	0.5	98.0	2.0
11	1.0	0.5	98.5	1.5
12	0.0	0.5	99.5	0.5
13	8.0	1.0	91.0	9.0
14	3.5	1.0	95.5	4.5
15	0.5	1.0	98.5	1.5
16	0.0	1.0	99.0	1.0
17	7.0	1.5	91.5	8.5
18	3.5	1.5	95.0	5.0
19	0.5	1.5	98.0	2.0
20	6.0	2.0	92.0	8.0
21	3.5	2.0	94.5	5.5
22	0.5	2.0	97.5	2.5
23	5.0	2.5	92.5	7.5
24	4.5	2.5	93.0	7.0
25	4.0	2.5	93.5	6.5
26	3.5	2.5	94.0	6.0
27	0.5	2.5	97.0	3.0
28	0.5	3.0	96.5	3.5
29	2.5	3.5	94.0	6.0
30	2.0	3.5	94.5	5.5
31	1.5	3.5	95.0	5.0
32	1.0	3.5	95.5	4.5
33	0.5	3.5	96.0	4.0
34	2.5	4.0	93.5	6.5
35	2.5	4.5	93.0	7.0
36	2.5	5.0	92.5	7.5
37	2.0	6.0	92.0	8.0
38	1.5	7.0	91.5	8.5
39	1.0	8.0	91.0	9.0
40	0.5	9.0	90.5	9.5
41	0.0	10.0	90.0	10.0

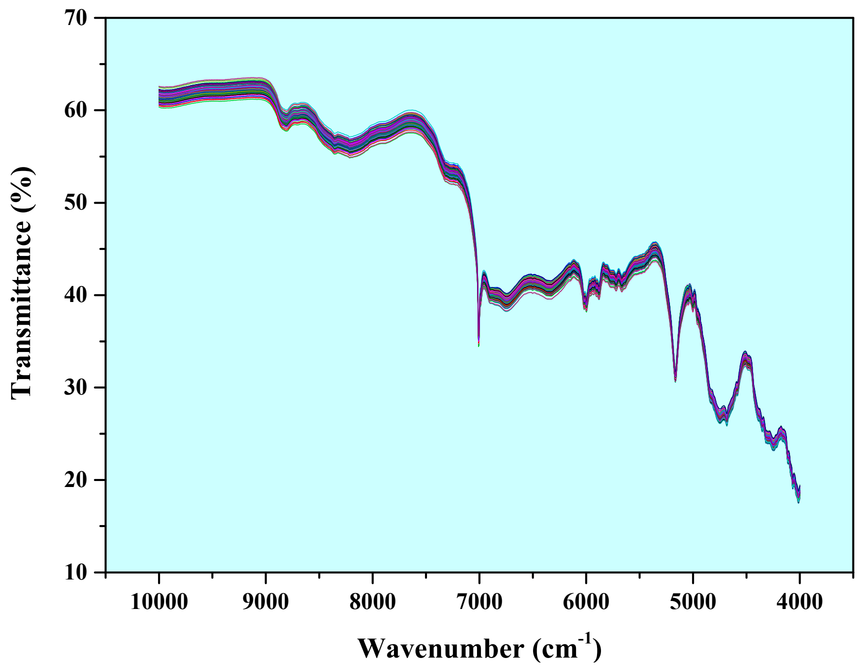

Figure S1. NIR spectra of the CFZ tablet samples.

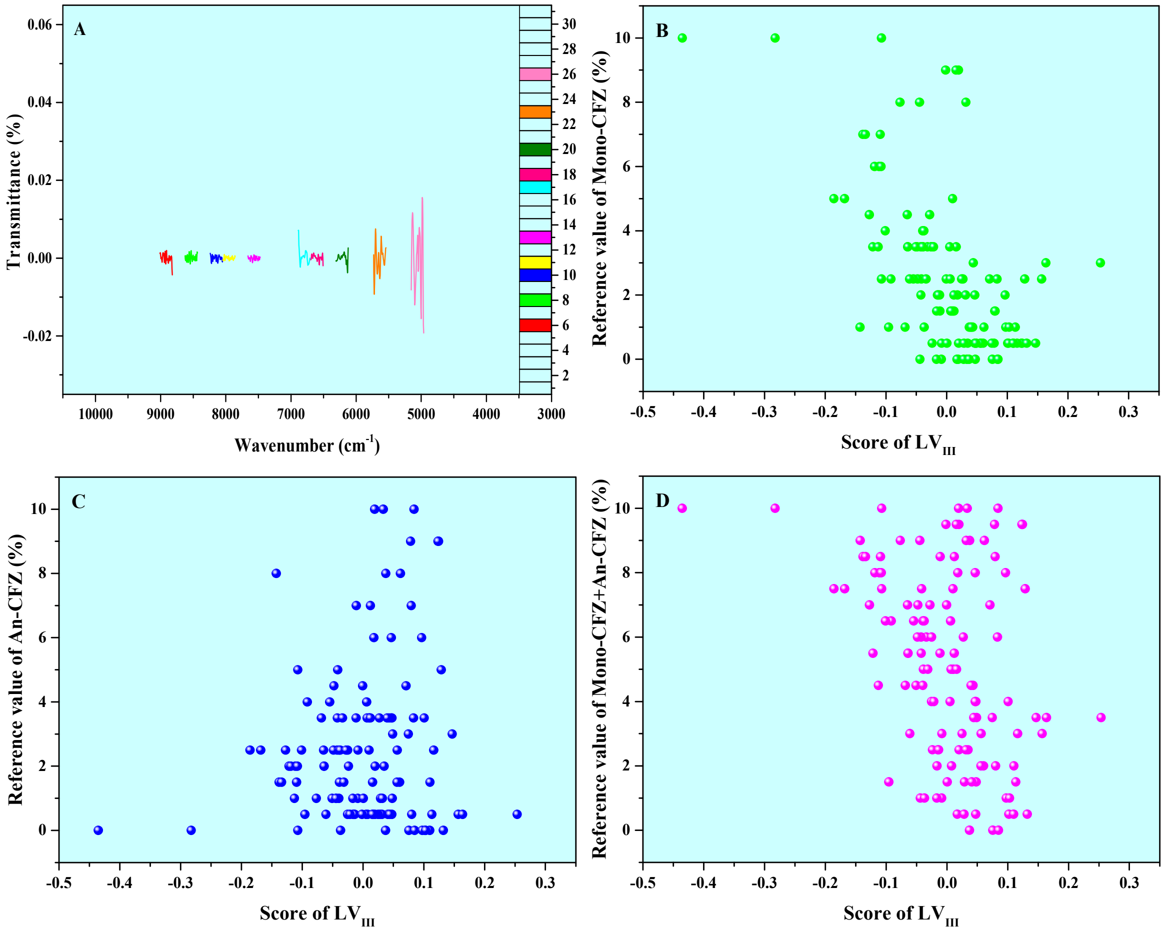

Figure S2. Loadings (A) and scores of LV_{III} (B, C, D). The color curves in A represented the ACO algorithm selected spectral bands, and the white represented the ACO algorithm not selected spectral bands. B, C and D represented the relationship between the scores of LV_{III} and the reference values of Mono-CFZ, An-CFZ and total polymorphic impurities (Mono-CFZ + An-CFZ) content, respectively.

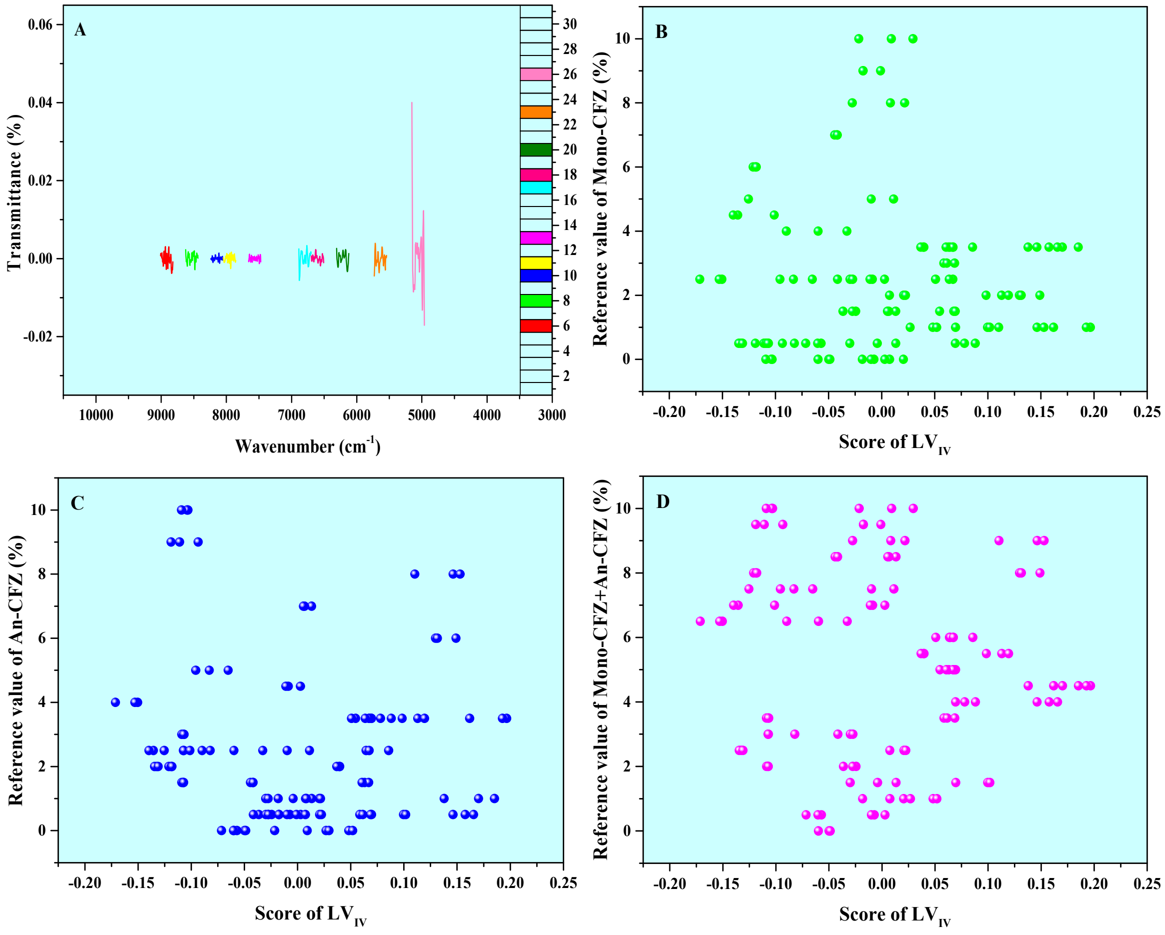

Figure S3. Loadings (A) and scores of LV_{IV} (B, C, D). The color curves in A represented the ACO algorithm selected spectral bands, and the white represented the ACO algorithm not selected spectral bands. B, C and D represented the relationship between the scores of LV_{IV} and the reference values of Mono-CFZ, An-CFZ and total polymorphic impurities (Mono-CFZ + An-CFZ) content, respectively.

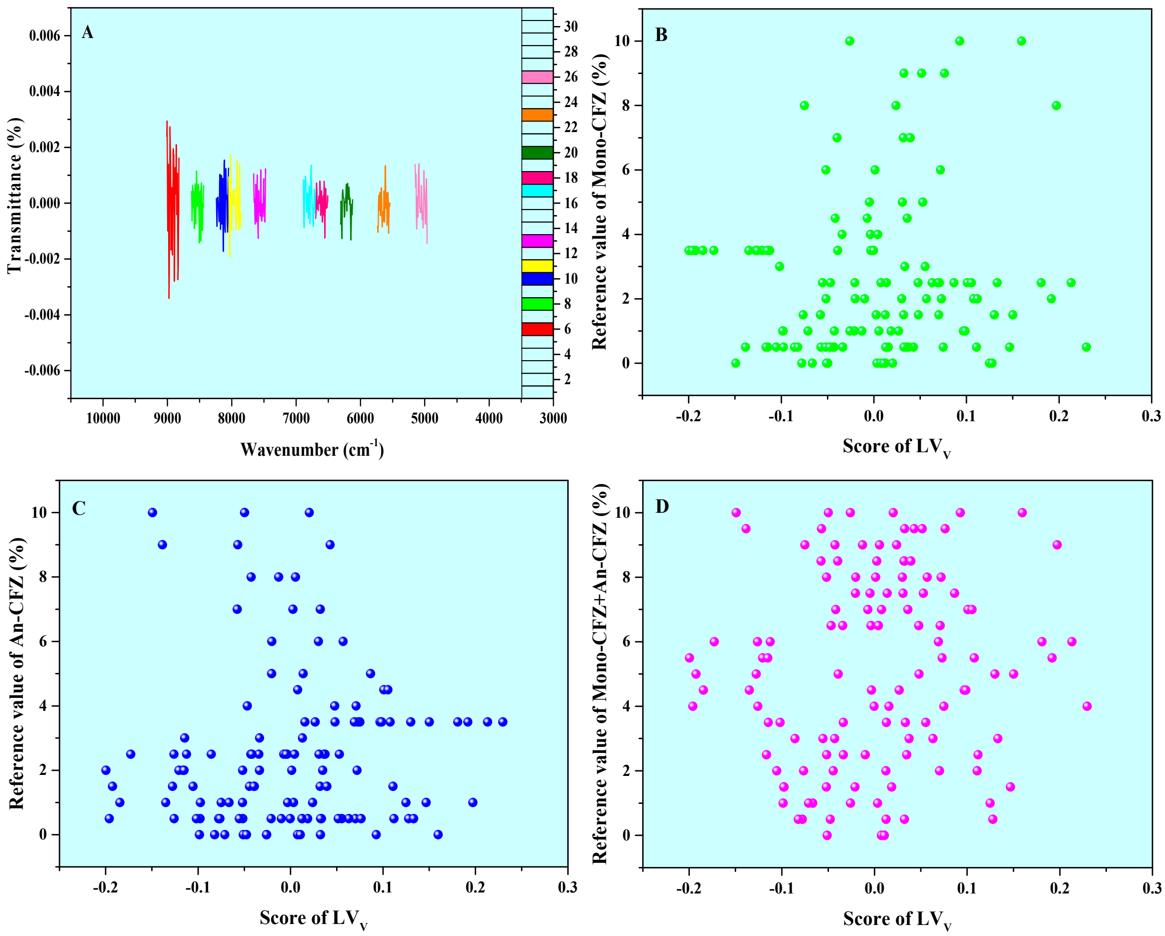

Figure S4. Loadings (A) and scores of LV_V (B, C, D). The color curves in A represented the ACO algorithm selected spectral bands, and the white represented the ACO algorithm not selected spectral bands. B, C and D represented the relationship between the scores of LV_V and the reference values of Mono-CFZ, An-CFZ and total polymorphic impurities (Mono-CFZ + An-CFZ) content, respectively.
